# Interaction of Stress, Lead Burden, and Age on Cognition in Older Men: The VA Normative Aging Study

**DOI:** 10.1289/ehp.0901115

**Published:** 2009-11-06

**Authors:** Junenette L. Peters, Marc G. Weisskopf, Avron Spiro, Joel Schwartz, David Sparrow, Huiling Nie, Howard Hu, Robert O. Wright, Rosalind J. Wright

**Affiliations:** 1 Department of Environment Health, Harvard School of Public Health, Boston, Massachusetts, USA; 2 Veterans Affairs Boston Healthcare System and Boston University Schools of Medicine and Public Health, Boston, Massachusetts, USA; 3 The Channing Laboratory, Department of Medicine, Brigham and Women’s Hospital, Harvard Medical School, Boston, Massachusetts, USA; 4 Department of Environmental Health Sciences, University of Michigan School of Public Health, Ann Arbor, Michigan, USA

**Keywords:** aging, blood, bone, lead, cognition, psychological stress

## Abstract

**Background:**

Low-level exposure to lead and to chronic stress may independently influence cognition. However, the modifying potential of psychosocial stress on the neurotoxicity of lead and their combined relationship to aging-associated decline have not been fully examined.

**Objectives:**

We examined the cross-sectional interaction between stress and lead exposure on Mini-Mental State Examination (MMSE) scores among 811 participants in the Normative Aging Study, a cohort of older U.S. men.

**Methods:**

We used two self-reported measures of stress appraisal—a self-report of stress related to their most severe problem and the Perceived Stress Scale (PSS). Indices of lead exposure were blood lead and bone (tibia and patella) lead.

**Results:**

Participants with higher self-reported stress had lower MMSE scores, which were adjusted for age, education, computer experience, English as a first language, smoking, and alcohol intake. In multivariable-adjusted tests for interaction, those with higher PSS scores had a 0.57-point lower (95% confidence interval, −0.90 to 0.24) MMSE score for a 2-fold increase in blood lead than did those with lower PSS scores. In addition, the combination of high PSS scores and high blood lead categories on one or both was associated with a 0.05–0.08 reduction on the MMSE for each year of age compared with those with low PSS score and blood lead level (*p* < 0.05).

**Conclusions:**

Psychological stress had an independent inverse association with cognition and also modified the relationship between lead exposure and cognitive performance among older men. Furthermore, high stress and lead together modified the association between age and cognition.

Cognitive decline has been associated with aging, and as the U.S. population shifts to a more elderly population, there is growing concern about the implications of cognitive dysfunction. However, cognitive decline varies widely across ages, which suggests that it may not be just a natural consequence of aging but may be linked to multiple risk factors (Wright et al. 2005).

The relationship between lead and cognitive impairment has been documented extensively in children and in occupationally exposed populations (e.g., Fiedler et al. 2003; Schwartz et al. 2001, 2005; Stewart et al. 1999; Winker et al. 2006). Previous studies by our group and others have also shown an inverse association in bone lead levels as well as blood lead levels with cognition and changes in cognition over time among nonoccupationally exposed older men and older women (Balbus-Kornfeld et al. 1995; Muldoon et al. 1996; Shih et al. 2007; Weuve et al. 2009). Levels of lead in blood represent acute exposure and levels in bone represent cumulative exposure.

Psychological stress (hereafter referred to as stress) has also been associated with decrements in short-term memory and attention (e.g., Levy et al. 1994; Mahoney et al. 1998; Vitaliano et al. 2005). However, stress itself is not uniformly negative (Cory-Slechta et al. 2008) and, under some conditions, may result in improved learning and memory (Zheng et al. 2007). In general, stressful events may result in negative emotional states, such as depression and anxiety, which in turn may exert lasting effects on physiologic processes that influence disease states or enhance vulnerability to other environmental factors (e.g., lead). The negative emotional response to life events (stressors) results when one perceives or appraises these events as overwhelming their ability to cope (Cohen et al. 1983; Lazarus and Folkman 1984). In response, physiologic systems may operate at higher or lower levels relative to normal homeostasis. The resulting long-term damage of unchecked accommodation of defensive processes (e.g., neural, immune, endocrine) is conceptualized as allostatic load (Lupien et al. 2007; McEwen 2007, 2008).

Exposure to both lead and stress often co-occur and potentially operate through overlapping biologic pathways of action [e.g., the hypothalamic–pituitary–adrenal (HPA) axis with disrupted release of glucocorticoids (e.g., cortisol). Recent laboratory studies have demonstrated that stress (restraint, cold, and novelty) modifies the neurotoxic effects of lead; moreover, lead and stress may have a combined effect in the absence of the effect of each alone (Cory-Slechta et al. 2008; Virgolini et al. 2005, 2008). Laboratory studies also show that the interactive effect is not limited to early development, a finding that indicates longer-term vulnerability (Agrawal and Chansouria 1989; Kim and Lawrence 2000). In a recent human study, Glass et al. (2009) found joint effects between neighborhood psychosocial hazards and cumulative lead exposure on cognitive function in older adults.

In this study, we cross-sectionally examined the modifying potential of psychological stress on the relation of cumulative and acute lead exposures as predictors of cognition in a cohort of older men from the Normative Aging Study (NAS). We previously reported an association between lead and cognition (Weisskopf et al. 2004, 2007; Wright et al. 2003) and an interaction of lead and age on cognition in this cohort (Wright et al. 2003). We hypothesized that high stress would lower the scores on the Mini-Mental State Examination (MMSE; Psychological Assessment Resources, Lutz, FL) and modify the lead-MMSE association and that the combined elevation of lead and stress would modify the relationship between age and cognitive impairment.

## Materials and Methods

### Study population

The NAS cohort is a longitudinal study on aging that was established in 1963 by the Veterans Administration (now the Department of Veterans Affairs); the subgroup of participants used in our analyses have been previously described (Cheng et al. 2001; Hu et al. 1996). Briefly, the NAS is a closed cohort of 2,280 male volunteers from the Greater Boston, Massachusetts, area. Men were screened at entry and enrolled if they had no chronic medical condition. Participants have been reevaluated every 3–5 years using questionnaires and detailed onsite physical examinations.

Cognitive testing was performed on 1,031 of the men still participating in the NAS between 1993 and 1997. Of these, 1,011 had a blood lead measure, 717 a bone lead measure, 838 a perceived stress measure, and 615 a stress appraisal measure, which is described below. Lead and questionnaire measurements were matched to the same year as the MMSE; however, if no questionnaire measurement was available for that year, the questionnaire data collected in the preceding evaluation cycle (within 3 years) were used. We used data reported up to 3 years before the MMSE test for 28 subjects. If no bone lead measure was available for the year of the participant’s MMSE score, we used the closest measure, within 2 years.

The present study was approved by the Human Research Committees of Brigham and Women’s Hospital and the Department of Veterans Affairs Boston Healthcare System, and written informed consent was obtained from subjects prior to participation.

### Cognitive assessment

The MMSE is a brief global examination of cognition that covers several domains including orientation to place and time, memory, attention, language, and ability to copy a design (Dufouil et al. 2000). We excluded the question on county (“What county are we in?”), as counties have little political significance in Massachusetts and are generally unknown to residents and thus of little diagnostic value. In this study, the maximum MMSE score was 29.

### Stress measures

Two measures of stress were available in the NAS. Using a health and social behavior questionnaire (Aldwin et al. 1996; Peters et al. 2007; Yancura et al. 2006), participants were asked to think of, list, and describe the most stressful thing that occurred to them in the past month. They were then asked, “Compared to other problems you might have had in the past, how stressful was this problem to you? (By stressful we mean how much it bothered or troubled you)”; participants rated this question on a 7-point scale. To facilitate the interpretability of interactive effects, the stress levels were dichotomized as low self-report of stress (≤ 5) and as high self-report of stress (> 5), as we had done in previous analyses (Peters et al. 2007). In other research, this measure was positively associated with a sense of threat and negative affect and negatively associated with a sense of challenge and positive affect supporting its construct validity (Yancura et al. 2006). In our subgroup, the measure correlated with a global distress index of the Brief Symptom Inventory (*r* = 0.21; *p* < 0.01) (Derogatis 1993) as well as with the Perceived Stress Scale (PSS) (*r* = 0.23; *p* < 0.01) (Peters et al. 2007).

The 14-item PSS (Cohen et al. 1995), a validated measure of stress appraisal, was also used to ascertain the degree to which respondents felt their lives were unpredictable, uncontrollable, and overwhelming to their coping resources in the month before the PSS was administered. Each item is scored on a 5-point scale that ranges from “never” (0) to “very often” (4); scores are obtained by summing the items. The PSS is the most widely used stress appraisal measure (Pizzagalli et al. 2007) with documented reliability and validity; it correlates with life events scores and depressive and physical symptomatology and has been shown to be a better predictor of a number of health outcomes compared with life-event measures of stress (Cohen et al. 1983, 1995). To facilitate interpretability of the interaction term, we dichotomized PSS by the median such that PSS ≤ 18 was characterized as low PSS and > 18 as high PSS; these values are consistent with prior studies (e.g., Kuiper et al. 1986; Pizzagalli et al. 2007).

### Lead measurement

Blood lead was analyzed using Zeeman background-corrected graphite furnace atomic absorption (ESA Laboratories, Chelmsford, MA, USA). The instrument was calibrated with Standard Reference Material 955a, lead in blood (National Institute of Standard and Technology, Gaithersburg, MD). Ten percent of the samples were run in duplicate, at least 10% as controls and 10% as blanks. When these samples were compared with reference samples from the Centers for Disease Control and Prevention, we found that the precision ranged from 8% for concentrations < 30 μg/dL to 1% for higher concentrations.

Bone lead was measured for 30 min each at the midtibia shaft and patella using a K-shell X-ray fluorescence instrument (ABIOMED, Inc, Danvers, MA). The tibia and patella have been used for bone lead research because they consist primarily of cortical and trabecular bone, respectively, with differing toxicity potential for each. Technical specifications and validity of this instrument are described in detail elsewhere (Burger et al. 1990; Hu et al. 1990, 1994). We excluded tibia and patella bone measures with estimated uncertainties > 10 μg/g and 15 μg/g of bone, respectively, because these measures usually reflect excessive patient movement during measurement (Hu et al. 1996)

### Analysis

We first assessed the relationship between each of the stress measures and the MMSE. We next considered the modifying effect of stress on age by fitting a model that included the main effects of stress and age plus an interaction term of stress times age predicting the MMSE score.

We then assessed the interactive relationship between lead and stress by testing a model that included the main effects of lead and stress plus an interaction term of lead times stress to predict MMSE score. We log-transformed the lead measures to address the influence of extreme values. We modeled the association by interquartile range (IQR) of log lead concentrations (approximately a 2-fold increase): blood lead (0.69 log units), patella lead (0.78 log units), and tibia lead (0.77 log units). We also checked our results by modeling untransformed lead values after using the extreme studentized deviation (ESD) many-outlier method to remove extreme outliers (Rosner 1983). Finally, we assessed the relationship of lead–stress combinations as modifiers of the relationship between age and MMSE score. For these analyses, we dichotomized lead measures by their median: 5 μg/dL for blood lead, 26 μg/g for patella lead, and 19 μg/g for tibia lead; and created the following four groups: high stress and high lead, high stress and low lead, low stress and high lead, and low stress and low lead. We then ran the analyses with the main effects of lead–stress groups and age and interaction terms of lead–stress group times age to predict MMSE score.

We ran the analyses using generalized linear models with SAS software (SAS Institute Inc., Cary, NC). The analyses were performed separately for the interaction of each lead measure (tibia lead, patella lead, and blood lead) and for each stress measure (self-report of stress appraisal and the PSS score). All analyses were adjusted for age (years), education (< 12 years, 12 years, 13–15 years, ≥ 16 years), smoking (never, former, current), alcohol intake (grams/day), computer experience (yes/no), and English as a first language (yes/no).

## Results

A total of 811 participants in the NAS who completed the MMSE also had a lead measurement (blood, patella, or tibia) and at least one stress measure (self-report of the most stressful life event or the PSS). Table 1 summarizes participant characteristics as well as stress, cognition, and lead measures. In bivariate analyses, none of the lead measures or covariates were associated with either stress measure. This was also true for the relationship of the covariates with lead measures.

The differences between those with and without bone lead measurements have been reported elsewhere (Weisskopf et al. 2004; Wright et al. 2003). For those with a measure of their most stressful event versus those without, we noted no differences in any covariate or lead measure except for education (those who provided this stress measure had slightly higher education).

The most stressful life event measure was significantly associated with a 0.44 lower [95% confidence interval (CI), −0.77 to −0.10] MMSE score. This is equivalent to the effect of 6.4 years of age in our data. Higher PSS scores were also associated with a 0.20 lower MMSE score, although this did not quite reach statistical significance (95% CI, −0.43 to 0.03). No interaction was observed with age for either the most stressful event score or the PSS.

**Figure 1:**
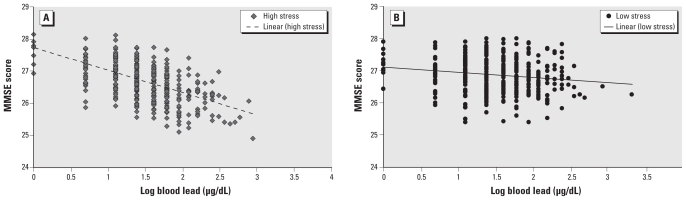
The relationship of log blood lead to predicted MMSE by high PSS (*A*) and by low PSS (*B*).

Both stress measures showed a trend toward negatively modifying the association of lead on cognition. Only the interaction between PSS and log blood lead was significant. Among men with higher PSS scores, an IQR increment in log blood lead was associated with a significant 0.57 lower (95% CI, −0.90 to −0.24) MMSE score, but among men with lower PSS scores, this same increase in log blood lead was associated with a nonsignificant 0.05 lower MMSE score (95% CI, −0.36 to 0.26), a −0.52 difference per IQR of log blood lead by stress (*p*-interaction = 0.02) (Table 2; Figure 1). A marginal negative interaction was also found between PSS and log patella lead (*p*-interaction = 0.06) (Table 2). In analyses of untransformed lead measurements excluding those identified as lead outliers (*n* = 7 for patella and *n* = 8 for blood) by the ESD procedure, we observed a stronger negative interaction between PSS and patella lead on the MMSE score (*p*-interaction = 0.02), although the interaction between PSS and blood lead was not significant (*p*-interaction = 0.23).

**Figure 2:**
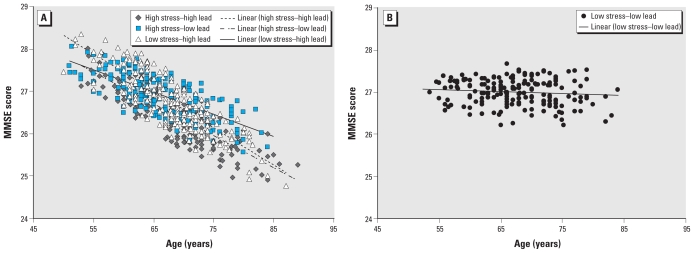
Relationship between age and predicted MMSE scores (*A*) by combined high or low PSS and high or low blood lead categories with at least one high on either or both and (*B*) by low PSS and low blood lead.

Based on the positive two-way interaction with PSS, we investigated the association of the relationship with age for each of the PSS–lead categories: high stress–high lead, high stress–low lead, and low stress–high lead compared with low stress–low lead (Table 3). For blood lead and PSS, none of the groups (high stress–high lead, high stress–low lead, low stress–high lead) differed from each other, but they differed significantly from the low stress–low lead group in their interaction with age to predict MMSE score (Figure 2). For each year increase in age, we noted that the participants in these stress–lead categories showed a significantly greater reduction in the MMSE score than did those in the low stress–low lead group. For the PSS–lead categories for patella lead, only the low stress–high lead group showed significant interactions with age.

## Discussion

In this cohort of older men, increased self-report of stress was related to lower cognition. Moreover, an inverse association with blood lead and MMSE was more pronounced among those who reported higher perceived stress using the PSS than among those who reported lower perceived stress. In addition, the combination of perceived stress and lead modified the relationship between age and cognition. This study corroborates laboratory studies and one other human study that indicated lead and stress interact to affect cognitive function (Cory-Slechta et al. 2008; Glass et al. 2009) and further supports the theory that cognitive impairment is not singularly a result of aging but due to risk factors working in concert.

Previous studies have reported that heterogeneity in cognition is especially pronounced in the elderly compared with younger adults (Lupien et al. 2005). Sapolsky et al. (1986) reported that stress exposure over the life course, likely mediated through disrupted stress hormones, significantly affect the aging process. Our group and others have reported a relationship between biomarkers of lead and cognition as well as a negative interaction between lead and age on cognition in older adults (Balbus-Kornfeld et al. 1995; Shih et al. 2007; Weuve et al. 2009). To our knowledge, this is among the first studies to assess the interaction of lead and psychological stress on cognition in older men and the first to investigate the combined association of lead and stress as a modifier of the relationship between age and cognition.

Aging has been associated with an increase in oxidative stress and elevated glucocorticoids (Pardon 2007; Sapolsky et al. 1986). It has also been associated with impaired plasticity of the HPA axis in experimental studies and appears to predict negative effects of stress (Lupien et al. 2007; Pardon 2007). The outcome of chronic stress and aging on brain function shows similarities; however, stress and aging seem to impact cognition via different underlying mechanisms (Pardon 2007). To add to the complexity, there is a paradox in stress–aging interactions: Although some evidence suggests vulnerability to stress can increase with age, other data indicate that the threshold of tolerance to stress may increase with age (Pardon 2007).

As proposed by Cory-Slechta and others (2008), the interactive effect of lead and stress may follow a multihit model. Lead and stress can both work through the activation of the HPA axis, which results in the release of a cascade of hormones such as cortisol. Disruption of the optimal balance of these stress hormones may enhance central nervous system vulnerability if they present insults on the same system of the brain via different mechanisms, overwhelming the ability of the system to maintain homeostasis (Cory-Slechta et al. 2008).

We observed some differences in the relationship between the two stress measures on cognition and their interactive relationship with lead. We found a significant negative relationship between the most stressful life event measure and MMSE score and a marginal negative relationship with PSS. In addition, even though the direction of the association was the same, only PSS showed significant interaction with lead in association with MMSE. The most stressful life event rating assesses a stressful event judged by the respondent to have a negative impact, whereas the perceived stress measures the individual’s perception of the current demands exceeding the ability to cope (Cohen et al. 1993). These measures, although correlated, may measure different constructs (i.e., may have independent relationships with disease risk and be mediated by different processes) (Cohen et al. 1993). In a study looking at the stress relationship with the common cold, Cohen et al. (1993) proposed that *a*) measures such as the life events measure may pick up acute or direct effects, whereas the PSS may be indicative of dispositional affect (i.e., an overall predictable way of responding to situations) and that *b*) the former may drive the development of symptoms, whereas the latter may be related to increased susceptibility.

We also found a difference between the interactive association of stress and lead among the measures of lead exposure: a significant interaction with blood lead, marginal interaction with patella lead, and no interaction with tibia lead. Of note, there were substantially fewer bone lead measures than blood lead. However, our results for blood lead remained significant after we restricted the blood analyses to those with only bone lead measurements (data not shown). In a previous study in this cohort that looked at the cross-sectional relationship between lead and elevated MMSE, Wright et al. (2003) observed a significant inverse relationship with blood and patella lead but not with tibia lead. These authors also found interactive relationships between blood and patella lead with age predicting elevated MMSE (Wright et al. 2003). The relationship with blood and patella lead is consistent with the theory that bone lead is chronically released into blood, that mobilization rate in aging differs by bone type, and that this mobilized lead contributes to an acceleration in cognitive decline (Wright et al. 2003). Stress has been found to mobilize bone lead stores in animals (Bushnell et al. 1979). In human studies, cortisol decreased mineral density and increased bone loss (Cetin et al. 2001; Dennison et al. 1999; Reynolds et al. 2005). Of interest, the effect of cortisol differs by sex and was observed to affect trabecular (e.g., patella) bone in older men (Dennison et al. 1999; Reynolds et al. 2005). The differential effect of cortisol by sex may partially explain significant results found by Glass et al. (2009) between neighborhood hazards and tibia lead (only tibia lead tested) on cognitive function in a mixed sample of adults. In addition, the contrasting results may reflect a difference in the measure of stress or the limitation of the MMSE used in our study to differentiate between domains of cognitive function that may be associated with different lead exposure measures (Weisskopf et al. 2007).

The combined effect of lead and stress is of particular concern, because HPA axis dysfunction has been linked to myriad disorders, in addition to cognitive impairment, including cardiovascular and metabolic diseases and psychiatric disorders (Cory-Slechta et al. 2008). Indeed, we showed the interactive association of lead and stress on blood pressure and the prospective risk of hypertension in this same cohort (Peters et al. 2007). It also is relevant in the context of low socioeconomic populations where the prevalence of these disorders is high and stress and lead tend to co-occur. Thus, the public health implications may be significant, given the possibility of improved neurobehavioral performance after reducing blood lead, which has been shown in serveral studies (Chuang et al. 2005; Schwartz et al. 2001; Winker et al. 2006), and stress (Bremner et al. 2008).

We note a number of limitations that may be addressed in future research. This study is cross sectional, so temporality cannot be established. It is conceivable that deficits in cognitive function could be a source of stress or produce stressful experiences. As eluded to earlier in the discussion, use of the MMSE may be considered a limited assessment of cognition; however, the strength of the MMSE is that it is a general measure that is widely used and understood. We evaluated relationships using two measures of psychological stress and three measures of lead exposure, raising the issue of multiple comparisons. However, we chose to make these comparisons because of reported differences between the stress and lead exposure measures in their relationship with disease. In addition, although we controlled for a number of risk factors, there is the risk of omitted or inadequately controlled confounders. In addition, this study was not conducted in a low socioeconomic population where there is a greater likelihood of dual exposure to stress and lead. Finally, there are noted differences in the association of stress with cognition and in the interactive effect among males and females (at least in laboratory studies) (Cory-Slechta et al. 2008; Virgolini et al. 2008; Wang et al. 2007). This finding suggests the need to look more closely at sex differences and the relationships found in our study.

In summary, our results show that stress is associated with lower cognition and modifies the relationship of age to cognition among community-dwelling older adult males. Furthermore, stress negatively modifies the relationship of blood lead and cognition, and combined high lead and high stress negatively modify the association of age with cognition.

## Figures and Tables

**Table 1:** Characteristics, stress levels, cognitive score, and lead exposure levels of subjects in the study population.

Variable	Measurements^a^	*n*
Age (years)	67.9 ± 6.99	811
Education, years (%)		
< 12	6.4	52
12	28.1	228
13–15	26.1	212
≥ 16	39.3	319
Smoking (%)		
Never	27.5	223
Former	66.7	541
Current	5.8	47
Alcohol (g/day)	13.2 ± 17.8	811
Computer experience (%)		
Yes	40.9	332
No	59.1	479
First language		
English (%)		
Yes	90.8	736
No	8.9	72
Stressful life event measure (%)^b^		
High	20.5	114
Low	79.5	443
PSS (%)^c^		
High	48.7	360
Low	51.3	379
MMSE scores	26.8 ± 1.67	811
Blood lead (μg/dL)	5.07 ± 2.76	808
Patella lead (μg/g)	29.8 ± 18.7	579
Tibia lead (μg/g)	21.3 ± 13.2	579

a Values are mean ± SD except where noted.

b High self-reported stress appraisal is > 5 on a 7-point scale.

c High PSS is divided by the median (> 18) of scores ranging from 0 to 56.

**Table 2:** Multivariable regression of the modifying potential of high stress on the relationship of log-transformed patella lead, tibia lead, and blood lead on MMSE^a^.

Variables	*n*	High stress β (95% CI)	Low stress β (95% CI)	*p*-Value interaction
Stressful life event measure				
Patella lead	410	−0.20 (−0.59 to 0.20)	0.00 (−0.21 to 0.21)	0.38
Tibia lead	407	−0.05 (−0.46 to 0.36)	−0.05 (−0.27 to 0.16)	0.99
Blood lead	552	−0.45 (−1.07 to 0.16)	−0.07 (−0.36 to 0.23)	0.26
PSS				
Patella lead	523	−0.19 (−0.44 to 0.06)	0.11 (−0.11 to 0.33)	0.06
Tibia lead	521	−0.14 (−0.38 to 0.10)	0.03 (−0.21 to 0.28	0.29
Blood lead	732	−0.57 (−0.90 to −0.24)*	−0.05 (−0.36 to 0.26)	0.02

a Parameter estimates are based on IQR increase in log blood lead (2.0-fold increase in blood lead), log patella lead (2.2-fold increase in patella lead), or log tibia lead (2.2-fold increase in tibia lead). Models were adjusted for age, education, smoking, alcohol intake, computer experience, and English as a first language.

* *p* < 0.05.

**Table 3:** Multivariable regression of the modifying potential of PSS–lead categories on the relationship of age and MMSE.

	Blood lead	Patella lead	Tibia lead
High stress–high lead			
β (95% CI)	−0.07 (−0.10 to −0.04)*	−0.06 (−0.11 to −0.02)*	−0.04 (−0.08 to 0.01)
*p*-Interaction	0.001	0.08	0.68
High stress–low lead			
β (95% CI)	−0.05 (−0.08 to −0.01)*	−0.05 (−0.09 to −0.01)*	−0.08 (−0.12 to −0.004)*
*p*-Interaction	0.02	0.27	0.07
Low stress–high lead			
β (95% CI)	−0.08 (−0.11 to −0.04)*	−0.09 (−0.14 to −0.04)*	−0.05 (−0.11 to −0.004)*
*p*-Interaction	< 0.001	0.02	0.36
Low stress–low lead			
β (95% CI)	0.01 (−0.02 to 0.05)	−0.01 (−0.06 to 0.03)	−0.02 (−0.07 to 0.02)
*p*-Interaction	(Referent)	(Referent)	(Referent)

Parameter estimates are based on 1-year increase in age; models were adjusted for education, smoking, alcohol intake, computer experience, and English as a first language. High lead is categorized as above median lead (median for blood lead is 5 μg/dL; for patella lead 26 μg/g, and for tibia lead 19 μg/g).

* *p* < 0.05.
